# A Critical Health Literacy Podcast to Counter Health Misinformation at Scale: Randomized Controlled Trial

**DOI:** 10.2196/78003

**Published:** 2026-01-28

**Authors:** Vanesa Mora Ringle, Amanda Jensen-Doss

**Affiliations:** 1 Department of Education and Human Services, College of Education, Lehigh University, 111 Research Drive, Bethlehem, PA, 18015, United States, 1 610-758-3257; 2 Department of Psychology, College of Arts and Sciences, University of Miami, Coral Gables, FL, United States

**Keywords:** critical health literacy, evidence-based practice, EBP, health misinformation, digital health intervention, public health education, randomized controlled trial, RCT, parents

## Abstract

**Background:**

Widespread misinformation and low critical health literacy pose major barriers to public health worldwide. Rapid, scalable, and evidence-informed digital interventions are urgently needed to strengthen the public’s ability to make informed health decisions.

**Objective:**

Informed by critical health literacy frameworks, we developed and tested a brief, story-based critical thinking podcast, *Parents Making Informed Health Choices*, that was designed to improve critical health literacy and decision-making among US parents.

**Methods:**

We conducted a 2-phase study. First, 5 parents participated in the user testing of the prototype podcast and provided qualitative feedback to refine content and delivery. The final podcast delivered 9 evidence-based practice principles through relatable scenarios about mental and physical health. In the second phase, we conducted a 2-arm randomized controlled trial (N=250) with a national online sample of US parents. Participants were randomly assigned to listen to either the critical thinking podcast (n=128, 51.2%) or a control podcast (n=122, 48.8%). There were no significant preintervention group differences except for age, which was controlled for in all analyses. Primary outcomes included critical thinking about health claims, intended health behaviors, attitudes toward evidence-based mental health practices, and treatment preferences.

**Results:**

On average, parents were aged 35 (SD 7.8) years; 49% (121/247) were female, 75% (185/248) were White; and 60.0% (148/248) had a bachelor’s degree or higher. Parents who listened to the critical thinking podcast demonstrated significantly improved critical thinking about health information compared to the control group (B=2.56; *P*<.001; ∆*R*^2^=0.06). They also reported stronger critical thinking–aligned intended behaviors (B=0.252; *P*=.001; ∆*R*^2^=0.015), and more evidence-informed treatment preferences (B=4.89; *P*=.038; ∆*R*^2^=0.02). The effect sizes were small to moderate across outcomes.

**Conclusions:**

Findings suggest that a brief, story-based digital podcast can meaningfully improve critical thinking about health information, intended behaviors, and evidence-based practice attitudes. Podcasts represent a promising, low-cost, and scalable strategy for promoting critical health literacy and countering health misinformation in the general public.

## Introduction

### Background

Health misinformation is now widely recognized as a public health threat [1]. During the COVID-19 pandemic and beyond, inaccurate health claims about vaccines and treatments have spread rapidly across digital platforms, undermining public trust and distorting health-related decision-making. Parents, in particular, face a deluge of conflicting health messages as they seek information about their children’s physical and mental health needs. However, few public health interventions directly aim to strengthen parents’ ability to critically assess health claims and make evidence-informed choices. Furthermore, scalable, low-intensity interventions that leverage popular media formats, such as podcasts, represent an underexplored avenue for enhancing critical health literacy at the population level. Accordingly, we developed and tested the effectiveness of a brief, story-based podcast intervention aimed at improving critical health literacy among US parents.

Many individuals lack the critical thinking skills necessary to make empowered and well-informed health choices [2,3]. Low critical thinking about health can lead to believing and acting on unreliable health information that is easily accessible by word of mouth, through mass media, and the internet [4-6]. Basing mental and physical health treatment decisions on unreliable information may lead to pursuing ineffective or potentially harmful services [7], delaying access to effective services (evidence-based practices [EBPs]), increasing risks, and wasting resources.

Unfortunately, few studies have focused on addressing the health-related critical thinking needs of the public, especially in the United States. Grounded in the Informed Health Choices Key Concepts framework, critical thinking about health extends beyond functional health literacy (ie, basic knowledge of health conditions and services) and involves applying EBP principles to access, understand, and evaluate sources of health information [8-10]. Studies across the world, even among high-income and highly educated populations, have found that the public engages in low levels of critical thinking about health [2,11-13].

Effective decision-making behavior in health care is complex, comprising not only critical thinking skills but also related attitudes and beliefs, among other characteristics and factors. Existing research has found that US adults and adolescents value scientific evidence when making health decisions; however, they have a limited understanding of what it means for practices to be evidence-based [14-16]. Additionally, US adults report that there are situations in which they would trust their doctor’s opinion over scientific research [17]. Issues of public trust in medicine and medical research are particularly prominent in relation to vaccines [18]. Indeed, despite their demonstrated efficacy, safety, and benefits, antivaccination or vaccine hesitancy attitudes persist and have resulted in adverse public health consequences (eg, increase in outbreaks of diseases that were under control or eradicated, such as measles) [18,19]. As such, examining critical thinking about health alongside attitudinal factors such as vaccine hesitancy is an important next step toward deepening our understanding of public health literacy and health decision-making.

Considering the potential impact of increasing the public’s ability to engage in critical thinking about health, it is crucial to develop and test brief, targeted, and scalable critical thinking interventions. This is especially important for parents, since they are responsible for health care decisions for both themselves and their children. Information and communication technologies play a crucial role in promoting critical health literacy by expanding access to information, fostering interactive learning environments, and enabling individuals to engage more actively with health content. Podcasts, in particular, offer a promising delivery method for addressing these challenges. They are widely accessible, cost-effective, and increasingly used for health communication and education [20-22]. As defined by the World Health Organization, digital health interventions are discrete uses of digital technologies, such as podcasts, to achieve health objectives [23]. Prior research and communication theory suggest that storytelling and narrative formats can improve engagement and promote behavior change, especially when combined with principles of EBP and tailored to target audiences [24]. However, few studies have tested the use of podcasts as a critical thinking intervention for health, and even fewer have done so using rigorous randomized designs.

Only one prior study has evaluated the effectiveness of such an intervention and focused on parents in Uganda [25]. Semakula et al [25] developed a 10-episode critical thinking podcast based on the needs of the Ugandan families. Via a randomized controlled trial (RCT) of 675 participants, the educational podcast was found to significantly improve parents’ critical thinking compared to those who only listened to a public service announcement. Moreover, improvements in critical thinking were observed across varying levels of educational attainment, including those who had only completed elementary education. Unfortunately, this intervention has yet to be adapted for and tested in the United States, and it solely focused on physical health issues, without any mental health examples.

While educating the public is essential across all areas of health care, it is especially urgent in the context of mental health, where treatment decisions are often complex and nuanced. Public discourse in this area is frequently shaped by unscientific or pseudoscientific claims, which can contribute to misinformation and reduce public trust in evidence-based care. This environment poses a significant barrier to individuals’ ability to make informed decisions and access appropriate mental health services [26-30]. Despite this, no existing critical health literacy intervention has specifically addressed decision-making in mental health care.

### This Study

The ability to critically think about health claims and treatment effectiveness enables the public to make well-informed health care decisions. By evaluating the effectiveness of treatments, the public can avoid potentially harmful or ineffective care and be empowered to take an active role in their health management. Thus, the primary aim of this study was to develop and test whether a brief critical thinking podcast increased critical thinking about health and related intended behaviors in comparison to an active control. The current intervention was adapted from the previously validated podcast used in Uganda [25] with cultural and contextual tailoring for a US-based audience. Additionally, because the original Ugandan podcast focused solely on physical health conditions, the current podcast expands its scope to include decision-making scenarios related to mental health. Given that this was the first evaluation of a critical health literacy podcast in the United States, the study also served as a proof-of-concept assessment to determine whether the approach would translate effectively in a different cultural and health communication context. A secondary aim was to examine the effects of the podcast on EBP attitudes, vaccine safety concerns, and mental health treatment preferences. We hypothesized that the critical thinking podcast would have a positive effect on all measured outcomes.

## Methods

### Design and Procedures

The study was conducted in the United States and included both community-based and online components. It consisted of two phases: (1) podcast development and user testing, and (2) podcast evaluation through an online RCT.

Podcast development included reviewing the scripts and storyboards of the Ugandan podcast by Semakula et al [25], and determining the needed adaptations (eg, changing examples about malaria). Communication theory on learning through entertainment informed the podcast design to ensure that messaging was not merely didactic, and there was a sense of relatability in the storylines and characters [24]. The first author and undergraduate research assistants scanned US mass media websites, such as Health News Review, for examples of mental health care claims relevant to US parents. For example, one of the episodes covers a claim regarding vaccines causing autism. Once the podcast script was finalized, it was produced by the Orange Umbrella, a production company housed within the university’s School of Communication.

We then conducted user testing where 5 parents from South Florida listened to the podcast prototype and provided feedback. A sample size of 5 is common in usability research where small samples are sufficient to identify most usability issues. For user testing, we recruited parents through the local chapter of the National Alliance on Mental Illness, advertisements in public community spaces, and Facebook posts. Eligible participants were aged at least 18 years, had at least 1 child aged less than 18 years, and were fluent in English. Participants were compensated with a US $15.00 gift card for their time and feedback. User-testing sessions lasted approximately 60 to 90 minutes, including 32 minutes of estimated individual podcast listening, a 20 to 30-minute semistructured interview, and a 5-minute online satisfaction survey. Their feedback was then reviewed and applied, and the podcast was finalized for testing through an online RCT. The podcast is available in SoundCloud [31].

A total of 250 parents participated in the RCT in May 2019 through Amazon Mechanical Turk (MTurk) [32,33]. A priori power analysis indicated that this sample size would provide adequate power to detect a large effect. As previously demonstrated by Jensen-Doss et al [33], MTurk is an appropriate space for recruiting parents online, especially for mental health care–related studies such as this one where capturing the perspectives of potential treatment seekers may be pertinent. The study was advertised to MTurk workers in the United States who had an MTurk approval rating of 98% or higher and were not participating in any other related study [3]. In the RCT, participants first provided consent and completed a baseline questionnaire of demographic and other characteristics. As in the original trial of the *Informed Health Choices* podcast by Semakula et al [25], outcome measures were completed after random assignment and after listening to the podcasts. Exposing participants to questionnaires prior to listening to the podcasts would have affected podcast listening, and it was expected that randomization would ensure that participants were equivalent at baseline (see the Randomization Success section). Following completion of baseline measures, participants were randomly assigned on a 1:1 ratio to either the critical thinking podcast or a control podcast. Participants in both conditions could not move on from the podcast listening page until 32 minutes (length of podcasts) had lapsed and they also could not fast forward. We also built in several “listening fidelity” checks (described in the Measures section). After they listened to the entire podcast, participants completed postintervention measures. Participants received a payment of US $7.25, in line with minimum wage at the time of the study. This study followed the CONSORT (Consolidated Standards of Reporting Trials) guidelines (Checklist 1).

### Podcast Intervention and Control Condition

The *Parents Making Informed Health Choices* podcast [31] consisted of nine 2 to 4-minute episodes, including an introduction episode and a conclusion episode. The entire podcast was 32 minutes long. The 7 main episodes covered 9 different EBP principles using medical and mental health conditions (eg, depression). Following the storylines developed for the Uganda *Informed Health Choices* podcast, the US podcast included 2 main characters who engaged in back-and-forth conversations while explaining and applying EBP principles to different medical and mental health conditions. Table 1 lists the EBP principles covered and provides examples of health care claims. The control podcast (a 33-minute-long mindfulness meditation) served as an inert condition that was similar in length to the critical thinking podcast, but included no content related to critical thinking.

**Table 1.** Evidence-based practice (EBP) principles [10] addressed in the *Parents Making Informed Health Choices* podcast. Examples are compared to those in the original *Informed Health Choices* podcast used in Uganda [25].

EBP principle	US claim examples	Uganda claim examples
Treatments should be compared.	Elderberry is an effective treatment for child flu.	Quail eggs make you very strong.
Treatments should be compared fairly.	Cognitive-mental therapy for youth anxiety and depression works.	Group support is helpful for someone who is depressed.
Findings from small studies can be misleading.	We can know that vaccines cause autism based on information from one small study.	Washing hands with soap does not stop children from getting diarrhea.
Association is not the same as causation^a^.	Contraceptive pills cause women to gain weight^a^.	A lot of women gain weight when they take contraceptive pills.
Expert opinion is not always right^a^.	Contraceptive pills cause women to gain weight^a^.	Eating some hot pepper will heal ulcers.
Anecdotes are unreliable evidence.	Butter can heal burns.	Putting cooking oil on a burn will heal it.
Treatments might be harmful^a^.	An herbal treatment for ADHD^b^ with no side effects exists^a^.	Quinine can cure malaria. It can also give you nausea and make you vomit.
Treatments have benefits and harms.^a^	Herbal treatment for ADHD with no side effects exists^a^.	Herbal medicines exist for malaria treatment that cure malaria and do not have any bad effects.
Common practice does not mean it is beneficial or safe.	Physical discipline is the best strategy for managing child behavior problems.	An herbal treatment called kyogero stops babies from getting infections.

a Covered within the same episode of the *Parents Making Informed Health Choices Podcast*.

b ADHD: attention-deficit hyperactivity disorder.

### Measures

#### Demographics and Other Characteristics

Participants’ age, gender, ethnicity, educational attainment, employment status, podcast listening habits or consumption (listening time in min), and other characteristics data (eg, treatment-seeking history) were collected.

#### Critical Thinking About Health

We assessed critical thinking about health using 18 items from the Claim Evaluation Tools, which consists of over 100 multiple choice questions that can be used with people aged 10 years and older [34,35]. We selected 18 questions that tapped into 9 different key concepts based on their cultural relevance to the United States and how well they performed in a validation study [34]. We also included 3 new questions that were worded exactly like Claim Evaluation Tools’ questions but replaced physical health conditions with mental health conditions. The overall critical thinking score was the number of correct responses out of 21 items. The internal consistency of the final 21-item critical thinking measure was good (α=.86).

#### Intended Behavior

Intended behavior related to critical thinking was assessed through 3 items created and used by Semakula et al [25]. The questions asked about the likelihood that someone will (1) find out what a treatment claim is based on, (2) find out if a claim is based on a research study comparing the treatment to no treatment (a fair comparison), and (3) saying “yes” if asked to participate in a research study comparing 2 treatments for an illness they have. Response options were on a 4-point Likert scale from very unlikely to very likely, and also included an “I don’t know” option.

#### Attitudes Toward EBPs in Mental Health

The Consumer Attitudes Towards Evidence Based Services Scale (CAEBS) [36] consists of 29 items that load onto 4 factors: (1) beliefs regarding therapists’ practices, (2) attitudes about mental health policy, (3) negative personal-level attitudes toward EBPs, and (4) negative societal-level attitudes towards EBPs. Items are rated on a 5-point scale from “strongly disagree” to “strongly agree.” Internal consistencies in this sample were as follows: factor 1, α=.77; factor 2, α=.59; factor 3, α=.88; and factor 4, α=.73. To increase reliability, we removed item 13 from the factor 2 scale (new α=.73), and item 24 from the factor 4 scale (new α=.80).

#### Attitudes Regarding Empiricism in Mental Health Treatment

We used 5 items created by Kirk et al [37] that assessed attitudes regarding empiricism in mental health treatment on a 5-point Likert scale, with higher scores indicating more agreement. The internal consistency of this 5-item scale in this sample was adequate (α=.77).

#### Treatment Preferences

We assessed treatment preferences using a measure developed by Kirk et al [37] that asked participants to rate how important various mental treatment components were to them by allocating 99 points across the following: (1) treatment being evidence-based, (2) therapeutic alliance, (3) therapist experience, (4) empathic qualities of the therapist, and (5) client speaking for the majority of sessions [37]. Participants were instructed that more points signify higher preference for that treatment component.

#### Vaccine Safety Concerns

Following prior studies [38,39], we examined vaccine safety concerns through three items: (1) “vaccines are unsafe,” (2) “vaccines have long-term negative side effects,” and (3) “If I had another infant today, I do not want him/her to get all the recommended vaccinations.” Participants rated these on a 5-point scale with higher scores indicating more negative vaccine attitudes. The internal consistency of this scale in this sample was α=.93.

#### Podcast Listening Fidelity

We assessed participant attention to the podcast through 7 questions about podcast content details that anyone who listened to the entire podcast should be able to answer, with specific questions for each study condition. Questions 1 to 6 were true or false, and question 7 required an open-ended response to a question about content at the end of the podcast. Responses to question 7 were assigned 0 to 2 points by the principal investigator; thus, participants could earn up to 8 points total.

#### Podcast Satisfaction

Participants in both conditions also rated their satisfaction with the podcast through 5 items that asked about overall satisfaction, likelihood of recommending to others, and relevance.

### Ethical Considerations

All procedures were approved by the University of Miami Institutional Review Board (20180596). The submitted protocol document was the one submitted to the institutional review board and received approval. There were no deviations from the protocol. Participants provided informed consent electronically prior to participating in the study. Data were collected anonymously through secure online surveys (Qualtrics) and analyzed in deidentified form. All data were stored on password-protected, encrypted servers to ensure participant privacy and confidentiality. The 5 user-testing participants were compensated with a US $15.00 gift card for their time and feedback. RCT participants received a payment of US $7.25, in line with minimum wage.

### Analytic Plan

Analyses were run using SPSS (version 28; IBM Corp). Descriptive statistics examined participant demographics, podcast satisfaction, and measures of data quality. We used multiple measures of data quality, including length of study participation (anticipating it would take at least 60 minutes), attention check questions, and 2 questions measuring consistency in the report of demographic information (age and youngest child age) strategically placed at the beginning and at the end of the study. Analyses checking randomization success examined differences in demographic variables between the 2 study conditions. We tested hypotheses about podcast effects using multiple linear regression for continuous variables and binomial logistic regression for categorical variables. The Cohen *d* values of 0.20, 0.50, and 0.80 indicated small, medium, and large effects, respectively; and *R*^2^ values of 0.01, 0.09, and 0.25 indicated small, medium, and large effects, respectively [40].

## Results

### Participants

Five parents participated in the user-testing phase of the study, and 250 parents participated in the online RCT. On average, parents in the RCT were aged 35 (SD 7.8) years with 49% (121/147) being female, 75% (185/248) being White, and 60.0% (148/248) having a bachelor’s degree or higher. Table 2 provides additional sample characteristics.

**Table 2.** Demographic characteristics of US parents who participated in the study’s user-testing and randomized controlled trial phases (N=250). Values in italics in the same row differed significantly (*P*<.05).

Characteristic	User-testing (n=5)	Online RCT^a^ (N=250)	Critical thinking podcast (n=128)	Control podcast (n=122)
Age (y), mean (SD; range)	36.40 (6.31; 30‐43)	34.98 (7.8; 20‐77)	*33.81 (7.5; 20-59)*	*36.20 (8.0; 23‐77)*
Sex (female)^c^, n (%)	5 (100)	121 (49.0)	59 (48.8)	62 (51.2)
Race or ethnicity^b^^c^, n (%)				
African American	0 (0)	41 (16.5)	19 (14.8)	22 (18.3)
American Indian or Alaska Native	0 (0)	2 (0.8)	1 (0.8)	1 (0.8)
Asian	1 (20)	13 (5.2)	4 (3.1)	9 (7.5)
Hispanic	2 (40)	35 (14.1)	14 (10.9)	21 (17.5)
White	3 (60)	185 (74.6)	101 (78.9)	84 (70)
Highest level of education achieved^c^, n (%)				
Some high school, no diploma	0 (0)	1 (0.4)	1 (0.8)	0 (0)
High school	0 (0)	29 (11.7)	14 (10.9)	15 (12.5)
Some college, no degree	0 (0)	44 (17.7)	22 (17.2)	22 (18.3)
Associate’s or technical degree	0 (0)	26 (10.5)	12 (9.4)	14 (11.7)
Bachelor’s degree	2 (40)	123 (49.6)	69 (56.1)	54 (43.9)
Master’s degree	2 (40)	23 (9.32)	10 (7.8)	13 (10.8)
Doctoral or other graduate degree	0 (0)	2 (0.8)	1 (0.8)	1 (0.8)
Employment status^c^, n (%)				
Currently working	5 (100)	213 (85.9)	115 (89.8)	98 (81.7)
Unemployed	0 (0)	9 (3.6)	3 (2.4)	6 (5)
Retired	0 (0)	2 (0.8)	1 (0.8)	1 (0.8)
Homemaker	0 (0)	22 (8.9)	8 (6.3)	14 (11.7)
Student or other	0 (0)	2 (0.8)	1 (0.8)	1 (0.8)
Annual household income^c^ (US $), n (%)				
Less than 19,999	—^d^	20 (8.0)	11 (8.6)	9 (7.5)
20,000 to 39,999	—	56 (22.4)	34 (26.6)	22 (18.3)
40,000 to 59,999	—	64 (25.6)	33 (25.8)	31 (25.9)
60,000 to 79,999	—	55 (22)	21 (16.4)	34 (28.4)
80,000 to 99,999	—	24 (9.6)	12 (9.4)	12 (10)
More than 100,000	—	29 (11.6)	17 (13.3)	12 (10)
Insured^c^, n (%)	—	220 (88)	114 (89.1)	106 (86.9)
Children (n), mean (SD; range)	2.20 (0.84; 1‐3)	1.73 (0.94; 1‐6)	1.69 (0.96; 1‐6)	1.77 (0.91; 1‐6)
Mental health history and service use^c^, n (%)				
Mental health diagnosis	—	109 (43.6)	52 (40.6)	57 (47.5)
Ever received psychotherapy for a psychological problem	—	114 (45.6)	54 (42.2)	60 (50.0)
Ever taken medication for a psychological problem	—	91 (36.4)	42 (32.8)	49 (40.8)
Podcast listening habits^c^ (min), n (%)				
<30	—	71 (28.4)	33 (25.8)	38 (31.4)
30‐60	—	84 (33.6)	43 (33.6)	41 (33.9)
61‐90	—	42 (16.8)	26 (20.3)	16 (13.2)
>90	—	52 (20.8)	26 (20.3)	26 (21.5 )

a RCT: randomized controlled trial.

b These percentages do not sum to 100 due to overlap across categories.

c Data missing (n=1-3).

d Not applicable.

### Analysis of Data Quality

**Figure 1.**
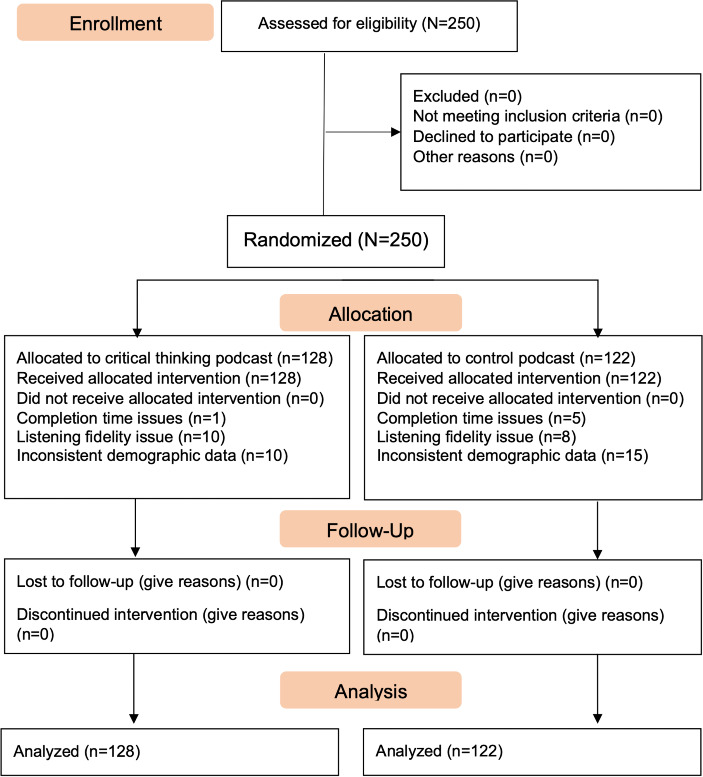
CONSORT (Consolidated Standards of Reporting Trials) diagram of participant flow through the *Parents Making Informed Health Choices Podcast* randomized controlled trial. This figure depicts participant flow for main study analyses where all randomized participants were retained in accordance with an intent-to-treat approach; exploratory post hoc analyses excluding 49 participants with data quality issues (indicated in the allocation section of the figure) are reported separately.

Out of 250 participants, 6 (2.4%) completed the study under 42 minutes (70% of the projected completion time); 18 (7.2%) failed attention check questions or obtained a score of 4 or lower on the listening fidelity measure; and 25 (10%) provided inconsistent demographic data. Consistent with an intent-to-treat approach, all randomized participants who completed study procedures were included in the primary study analyses (Figure 1). For completeness, we report exploratory post hoc analyses excluding the 49 participants with data quality issues at the end of this section. Parents in the critical thinking podcast condition obtained an average score of 6.73 (SD 1.68; range 1‐8) on the listening fidelity test and parents in the control condition earned an average score of 6.76 (SD 0.84; range 1‐8).

### Randomization Success

There were no significant differences between the critical thinking podcast condition and control condition at preintervention based on gender (*χ*^2^_1_=0.679; *P*=.41), education level (*χ*^2^_1_=0.88; *P*=.35) annual household income (*t*_248_=0.720; *P*=.47), podcast listening habits (*t*_248_=–0.744; *P*=.46), and podcast listening fidelity scores (*t*_248_=0.155; *P*=.88). However, the control group was significantly older, by 2 years (*t*_248_=2.43; *P*=.02); therefore, we controlled for age in all analyses. Given some variability and to reduce the possibility that significant relationships were due to third variables, we also included education and listening fidelity as control variables in outcome analyses. Table 3 includes descriptive statistics for study measures, including means and SDs for this sample.

**Table 3.** Descriptive data for study variables by condition and overall sample (N=250). Values in italics in the same row differed significantly at *P*<.05 based on regression analyses controlling for age, education, and listening fidelity.

Variable	Overall (N=250)	Critical thinking podcast (n=128)	Control podcast (n=122)
Critical thinking—continuous, mean (SD; range)	14.36 (5.41; 2‐21)	*15.48 (5.19; 2-21)*	*13.19 (5.42; 2‐21)*
Critical thinking—dichotomous, n (%)			
High score (1=18 or more correct out of 21)	99 (39.6)	*67 (52.3)*	*32 (26.2)*
Low score (0=17 or less correct out of 21)	151 (60.4)	*61 (47.7)*	*90 (73.8)*
Intended behavior (1-4), mean (SD; range)			
Find out what a claim was based on	3.53 (0.71; 1‐4)	3.54 (0.73; 1‐4)	3.52 (0.70; 1‐4)
Find out if a claim was based on a fair comparison study	3.61 (0.63; 1‐4)	*3.72 (0.55; 1-4)*	*3.49 (0.69; 1‐4)*
How likely are you to participate in a fair comparison study	3.08 (0.87; 1‐4)	3.13 (0.85; 1‐4)	3.03 (0.88; 1‐4)
EBP^a^ attitudes (1-5), mean (SD; range)			
Beliefs regarding therapists’ practices	24.74 (3.54; 10‐30)	24.85 (3.57; 13‐30)	24.61 (3.51; 10‐30)
Attitudes about mental health policy	13.40 (3.34; 5‐20)	13.65 (3.19; 6‐20)	13.14 (3.48; 5‐20)
Negative personal-level attitudes towards EBPs	23.97 (7.82; 9‐45)	*23.01 (7.43; 9-41)*	*24.98 (8.11; 9‐45)*
Negative societal-level attitudes toward EBPs	14.48 (3.47; 5‐20)	14.36 (3.51; 5‐20)	14.60 (3.44; 4‐20)
Empiricism attitudes (1-5)	4.08 (0.63; 2.2‐5)	4.15 (0.60; 2.4‐5)	4.01 (0.66; 2.2‐5)
Vaccine safety concerns (1-5)	6.1 (3.58; 3‐15)	6.03 (3.57; 3‐15)	6.12 (3.60; 3‐15)
Treatment preferences, mean (SD; range)			
Therapeutic alliance	24.66 (16.14; 0‐99)	23.57 (16.51; 0‐99)	25.80 (15.73; 0‐99)
Scientific studies show therapy is highly effective	28.20 (18.82; 0‐99)	*30.02 (20.55; 0-99)*	*26.29 (16.70; 0‐89)*
Therapist experience	16.33 (10.63; 0‐49)	16.02 (11.04; 0‐49)	16.66 (10.21; 0‐48)
Empathic therapist	18.39 (12.74; 0‐71)	17.77 (12.53; 0‐70)	19.05 (12.97; 0‐71)
Client speaking majority of session	11.42 (10.04; 0‐52)	11.63 (10.90; 0‐52)	11.20 (9.09; 0‐42)
Podcast satisfaction, mean (SD; range)			
Overall satisfaction (1-5)	4.18 (0.89; 1‐5)	4.16 (0.93; 1‐5)	4.20 (0.85; 1‐5)
Continue listening (1-5)	3.61 (1.16; 1‐5)	3.56 (1.19; 1‐5)	3.65 (1.14; 1‐5)
Recommend to others (0‐10)	6.71 (2.89; 0‐10)	6.76 (2.79; 0‐10)	6.66 (3.01; 0‐10)
Relevance to mental health questions (1-4)	2.97 (0.94; 1‐4)	3.05 (0.95; 1‐4)	2.88 (0.92; 1‐4)
Relevance to physical health questions (1-4)	2.80 (1.03; 1‐4)	3.14 (0.89; 1‐4)	2.43 (1.04; 1‐4)

a EBP: evidence-based practice.

### Effects on Critical Thinking

As hypothesized, parents who listened to the critical thinking podcast performed significantly better on the critical thinking measure than those who listened to the control podcast (*B*=2.56; *P*<.001), with a small-medium effect size (∆*R*^2^=0.06). Table 4 presents all results from linear regression analyses.

**Table 4.** Results from linear regression analyses examining critical thinking podcast effect on critical thinking, intended behavior, attitudes, treatment preferences, and overall podcast satisfaction (control variables: age, education, and listening fidelity).

Dependent variable	Critical thinking		
	B	SE	*β*
Critical thinking	2.56	0.515	.237^a^
Intended behavior			
Find out what a claim was based on	0.042	0.092	.029
Find out if a claim was based on a fair comparison study	0.252	0.078	.201^b^
How likely are you to participate in a fair comparison study	0.109	0.116	.063
EBP^c^ attitudes			
Beliefs Regarding Therapists’ Practice	0.073	0.093	.050
Attitudes About Mental Health Policy	0.495	0.416	.074
Negative Personal-Level Attitudes towards EBPs	–1.96	0.893	–.127^d^
Negative Societal-Level Attitudes towards EBPs	–0.160	0.446	–.023
Attitudes regarding empiricism	0.156	0.080	.124
Vaccine safety concerns	–0.185	0.400	–.026
Treatment preferences			
Therapeutic alliance	–1.82	2.08	–.056
Scientific studies show therapy is highly effective	4.89	2.34	.131^d^
Therapist experience	–1.50	1.33	–.071
Empathic therapist	–1.59	1.64	–.063
Client speaking majority of session	0.024	1.26	.001
Overall podcast satisfaction	–0.050	0.113	–.028

a *P*<.001.

b *P*<.01.

c EBP: evidence-based practice.

d *P*<.05.

### Effects on Intended Behavior

Compared to control parents, parents in the critical thinking podcast condition reported a significantly higher likelihood of “finding out if a claim was based on a fair comparison study” in the future (*B*=0.252; *P*=<.001; ∆*R*^2^=.04). There were no significant differences between the conditions regarding the likelihood of engaging in the other 2 intended behaviors assessed: “finding out what a claim was based on,” and “participating in fair comparison study.”

### Effects on Attitudes

Between-group differences in EBP attitudes were examined across the 4 attitudes factors of the CAEBS. Parents in the critical thinking condition had lower scores on the negative personal-level attitudes towards EBPs scale (*B*=−1.96; *P*<.05; ∆*R*^2^=0.015). There were no significant differences between podcast groups in the 3 other CAEBS EBP attitudes scales, attitudes regarding empiricism in mental health, or vaccine safety concerns.

### Effects on Treatment Preferences

Parents who listened to the critical thinking podcast expressed a significantly higher preference for *“*receiving effective therapies backed by scientific studies*”* compared to the control group (*B*=4.89; *P*<.05; ∆*R*^2^=0.02). All other mental health treatment preferences did not vary significantly based on condition.

### Post Hoc Analyses Without Intent to Treat Sample

We also ran analyses excluding the 49 participants who failed data-quality checks. When we excluded these individuals in the comparison analysis of parents’ critical thinking postintervention, we found that the magnitude of the intervention effect on critical thinking increased from 0.06 to 0.11 (*B*=0.525; *P*<.001). In regard to other outcomes, when we removed the intent to treat sample, we found that parents who listened to the critical thinking podcast reported significantly more positive attitudes regarding empiricism in mental health treatment (*B*=0.210; *P*<.05; ∆*R*^2^=0.03), and no longer found a significant difference between podcast groups in the negative personal-level attitudes towards EBPs scale.

## Discussion

### Principal Findings

To address the public health misinformation crisis at scale, we developed and tested the efficacy of a story-based, educational podcast—the *Parents Making Informed Health Choices Podcast*—a brief, scalable, low-intensity intervention designed to increase critical thinking about health among US parents. Through an online RCT, we found that listening to the podcast improved parent critical thinking and had an effect on intended behaviors, attitudes, and treatment preferences. To our knowledge, this is the first online RCT of a mass media, brief critical thinking intervention for a US audience.

A previous study’s findings of low levels of critical thinking among US parents and young adult college students demonstrated a need for a scalable critical thinking intervention for the US public [3]. The impacts on critical thinking demonstrated in this study add to the growing body of empirical evidence demonstrating the efficacy of critical thinking learning resources designed for the lay public [41-43]. The critical thinking effects observed in this US sample are similar to those observed in the original Ugandan podcast, where they also found a significant difference in postintervention critical thinking performance favoring parents in the critical thinking condition [25].

Out of the 3 intended behaviors assessed, parents who listened to the critical thinking podcast were more likely to report intending to find out if a treatment claim is based on a fair comparison study with control parents, but contrary to the hypotheses, these 2 other intended behaviors were not significantly different between the 2 study conditions. A possible explanation for this may be that the podcast directly and repeatedly mentions “fair comparisons,” whereas the other intended behaviors are less frequently mentioned. These findings also differ from the Ugandan podcast RCT, where they did not find any significant behavioral intention differences between study conditions.

In terms of attitudinal measures related to critical thinking about health, parents who listened to the critical thinking podcast had less negative personal-level attitudes toward EBPs (as measured by 1 of the 4 factors of CAEBS) [36], and reported a higher preference for receiving effective therapies backed by scientific studies. Contrary to expectations, conditions did not differ on any other EBP attitudes or vaccine safety concerns. Notably, this study was conducted prior to the COVID-19 pandemic; as such, public attitudes regarding vaccines may have changed.

Of the 4 CAEBS factors regarding consumer EBP attitudes (beliefs regarding therapists’ practices, attitudes about mental health policy, negative personal-level attitudes toward EBPs, and negative societal-level attitudes towards EBPs), the personal-level attitudes toward EBP factor is most in line with the content of the critical thinking podcast, with items such as “I don’t feel comfortable making treatment decisions.” Nevertheless, the null findings regarding 3 out of the 4 CAEBS scales were surprising given the general similarity between the concepts covered in the critical thinking podcast and the factors assessed by the CAEBS [36]. Upon closer scrutiny of the CAEBS’ scales and items, possible explanations become apparent. First, while the *Parents Making Informed Health Choices Podcast* centers on EBP principles and making them accessible to the lay public, it rarely mentions “evidence-based” terms because the focus is on teaching a skill rather than *telling* the audience they should seek out EBPs. On the other hand, the CAEBS frequently and explicitly mentions the term “evidence-based” and defines it in the instructions. Perhaps this suggests that evidence-based health care proponents and researchers have some middle ground to reach in regards to balancing efforts to teach *science and health literacy–based skills* (ie, critical thinking about health) and increasing consumer knowledge of *health care terms*. It would be interesting for future studies to examine whether explicitly and repeatedly mentioning EBPs in the podcast has a different effect on EBP attitudes as measured by the CAEBS. Another potential explanation for the null findings is that CAEBS items have a strong emphasis on society and policy themes, whereas the critical thinking podcast solely focused on individual actions around EBP principles.

It should be noted that the *Parents Making Informed Health Choices Podcast* achieved these effects through an online, one-time, brief, audio intervention whereas the original Ugandan podcast RCT used a lengthier podcast listening procedure. Thus, our choosing to prioritize ecological validity based on how the public interacts with health information on the internet did not diminish the intervention’s effects. Notably, parents in this study were tested on their critical thinking abilities and other factors a few minutes after listening to the podcasts. As such, a follow-up study is necessary to examine long-term effects.

Future studies should also determine who is most likely to benefit from these critical thinking interventions. For example, including a sample with a broader range of levels of educational attainment could help determine if education is a moderator. Another important future direction for critical thinking research is how to incorporate pragmatic tips on how parents can carry out critical thinking in conversations with health care providers, especially in situations where the evidence base may not be strong, but where a treatment is still recommended either because it is the only option or for other valid reasons. There are EBP principles that address these realities (eg, EBP principle “how certain is the evidence?”) [44], and future intervention or podcast development should focus on creating additional short episodes that capture these EBP principles in decision-making (eg,[45].

Furthermore, critical thinking interventions could complement a shared-decision making framework [46,47] in that critical thinking interventions encourage patient activation. Thus, future studies might consider combining or embedding critical thinking interventions within shared decision-making frameworks. Indeed, although this is not directly communicated in the *Parents Making Informed Health Choices Podcast*, characters in the stories often do model appropriate interactions with health care providers; however, the storylines do not conclude in a shared decision about health care services.

### Limitations

As with any investigation, this study has several limitations that suggest additional directions for future research. First, although online-convenience samples of parents generally provide reliable data [33] and this study sample was relatively diverse, especially in regard to socioeconomic status, they may not always be representative of all US parents, especially of parents in low-resource, community settings. Thus, future studies should focus on testing the efficacy of the podcast with more representative samples of parents from community settings that could greatly benefit from interventions such as this one. Along those lines, the *Parents Making Informed Health Choices Podcast* is currently only available to English-speaking parents, creating a disparity for parents who speak other languages, especially predominantly Spanish-speaking parents, who make up the fastest growing linguistic population in the United States. Future efforts should focus on translating these materials and testing their efficacy in other languages, especially Spanish. Some of this work is already starting with children in Spain [48]. Second, we have found evidence of only the very short-term efficacy of the *Parents Making Informed Health Choices Podcast*. Therefore, an important future direction is to conduct a long-term follow-up assessment of critical thinking with parents who participated in this online RCT, especially given that a 1-year follow-up study on the effects of the Ugandan critical thinking podcast found a decline in critical thinking abilities [49]. Finally, all outcome variables were about parent attitudes and preferences regarding mental health services for themselves, but we did not specifically ask about their attitudes and preferences in regards to mental health services for their children, which may be an important addition to future studies.

### Conclusions

This pilot trial provided empirical evidence regarding the efficacy of a brief, story-based critical health literacy podcast for US parents—a population frequently targeted by health misinformation. We found that listening to a brief podcast significantly improved parents’ critical thinking about health, as well as increased self-reported intended behavior, positive attitudes toward EBPs, and preference for EBPs. By delivering accessible, engaging content in a familiar digital format, podcasts may represent a scalable public health strategy to promote evidence-based decision-making and strengthen critical health literacy. Future research should examine long-term effects, implementation in diverse communities, and potential integration into broader digital health and health education ecosystems.

## Supplementary material

10.2196/78003 Checklist 1 CONSORT checklist.

## Abbreviations

CAEBS: Consumer Attitudes Towards Evidence Based Services Scale
CONSORT: Consolidated Standards of Reporting Trials
EBP: evidence-based practice
MTurk: Mechanical Turk
RCT: randomized controlled trial
